# Correlate factors and nomogram model establishment for women with low bone mineral density: a cross-sectional diagnostic prediction model study

**DOI:** 10.3389/fpubh.2026.1969424

**Published:** 2026-10-09

**Authors:** Xiaoli Yan, Yu Wang, Yang Wang, Qingshuai Yu, Chuan Zhang

**Affiliations:** 1 Health Management Center, The First Affiliated Hospital of Chongqing Medical University, Chongqing, China; 2 Department of Outpatient, The First Affiliated Hospital of Chongqing Medical University, Chongqing, China; 3 Department of Orthopedics-Spine Surgery Center, The Second Affiliated Hospital of Chongqing Medical University, Chongqing, China; 4 Department of Cardiology, The First Affiliated Hospital of Chongqing Medical University, Chongqing, China

**Keywords:** low bone mineral density, nomogram, prediction model, risk factors, women

## Abstract

**Background:**

The proportion of women suffering from osteopenia is high, and early diagnosis and treatment are crucial. However, clinical diagnosis requires special equipment and may involve radiation damage. The purpose of this study was to establish a prediction nomogram based on easily obtainable routine physical examination indicators and risk factors for the early prediction of low bone mineral density (LBMD) in women.

**Methods:**

We conducted a two-center cross-sectional diagnostic prediction model study involving women who underwent medical examinations from January 1, 2019, to December 31, 2021. Included participants (*n* = 3,121) from the physical examination center were randomly divided into a training set (2185) and an internal validation set (936) at a 7:3 ratio. Female outpatients (*n* = 1,138) from another independent medical center within the same period were enrolled as the external validation set. Dual-energy X-ray absorptiometry (DXA) was used to measure bone mineral density (BMD) at the lumbar spine (L1–L4) and left femoral neck. Demographic characteristics, laboratory test results, and DXA measurements were retrospectively extracted from the hospital electronic health record system. Univariate analysis and multiple logistic regression analysis were used to screen for risk factors related to LBMD, and a predictive model for LBMD was constructed with the training set. Afterwards, a nomogram was developed and validated by receiver operating characteristic (ROC) curve analysis, calibration curve analysis, and clinical decision curve analysis (DCA) in the internal validation set and the external validation set.

**Results:**

A total of 4,259 people were included in this study. The detection rates of LBMD in the training set, internal validation set, and external validation set were 30.48% (666/2185), 31.08% (291/936), and 33.48% (381/1138), respectively. The important relevant factors for LBMD in women were age (OR = 1.204, 95% CI: 1.184–1.225, *p* < 0.001), weight (OR = 0.943, 95% CI: 0.927–0.960, *p* < 0.001), serum creatinine (OR = 0.984, 95% CI: 0.970–0.999, *p* = 0.034), serum phosphorus (OR = 0.698 per 0.1 mmol/L; 95% CI: 0.641–0.761, *p* < 0.001) and serum magnesium (OR = 0.600 per 0.1 mmol/L; 95% CI: 0.486–0.741, *p* < 0.001). A nomogram was established as a tool for predicting LBMD based on the above relevant factors. The area under the ROC curve (AUC) of the nomogram was 0.894 (95% CI, 0.877–0.912) based on the training set. The calibration curve and DCA both indicated a high degree of accuracy in the model’s predictions in the internal and external validation sets.

**Conclusion:**

This novel nomogram can effectively predict LBMD in women, and the indicators are easy to obtain. This is helpful in screening high-risk populations for LBMD and early acceptance of personalized intervention measures.

## 1 Introduction

Osteoporosis is a common bone disease characterized by low bone mass and deterioration of bone tissue, leading to increased bone fragility and a greater risk of fractures, particularly in the hip, spine, and wrist (1). Approximately 200 million people worldwide suffer from osteoporosis (2), which causes clinical and economic burdens. Women are more susceptible to bone loss, especially after menopause, due to hormonal changes that affect bone density. A retrospective clinical study conducted in Sweden included individuals aged 50 years and older. Osteoporotic fracture was defined as at least one confirmed record of hip, humeral, forearm, or vertebral fracture in the Swedish National Patient Register (SNPR) from 1998 to 2012. A total of 362,899 cases were registered, with 96,847 in men, 266,052 in women, and an incidence rate of approximately 20% in women (3). As reported by the International Osteoporosis Foundation (IOF), the lifetime probability of developing osteoporotic fractures among women aged 50 years and above reaches 46.4% (4).

It is important to note that osteoporosis is often underdiagnosed because it is a “silent” disease, meaning it progresses without obvious symptoms until a fracture occurs (5); therefore, regular examinations are necessary. According to the World Health Organization (WHO) classification criteria (6, 7), dual-energy X-ray absorptiometry (DXA) is considered the gold standard diagnostic test for osteoporosis. The diagnosis is typically based on a T score of −2.5 or lower, which indicates reduced bone mineral density (BMD) and the presence of osteoporosis. Additionally, the WHO criteria define osteopenia (a preclinical stage of osteoporosis) as a T score between −2.5 and −1.0 – a stage that shares similar pathophysiological mechanisms with osteoporosis (e.g., impaired bone metabolism, disrupted bone microstructure) and exhibits a clear progression risk. Gourlay et al. (8) followed 3,702 women with osteopenia and reported that the transition time from early-stage osteopenia (*T* score −1.5 to −1.0) to osteoporosis was 17 years, from middle-stage osteopenia (*T* score −2.0 to −1.5) to osteoporosis was 5 years, and from late-stage osteopenia (*T* score −2.5 to −2.0) to osteoporosis was only 1 year. This highlights that osteopenia is not an isolated condition but a critical precursor to osteoporosis, and neglecting this stage will miss the optimal window for early intervention. Nevertheless, DXA are generally scarce in developing countries and economically disadvantaged areas. Combined with the financial burden on local residents, standardized bone mineral density examinations remain inaccessible to most populations. To enhance the efficiency and generalizability of osteoporosis screening, a variety of risk assessment tools have been developed for population-based preliminary screening, such as the IOF One-Minute Test, Osteoporosis Self-Assessment Tool for Asians (OSTA), Osteoporosis Risk Assessment Instrument (ORAI), Simple Calculated Osteoporosis Risk Estimation (SCORE), Osteoporosis Index of Risk (OSIRIS), and Osteoporosis Prescreening Risk Assessment (OPERA) (9–13). These easy-to-operate tools allow the general population to rapidly complete preliminary self-evaluation of osteoporosis risk. Nevertheless, they suffer from three drawbacks when applied to the early screening of subclinical bone loss, which underscores the urgent need to establish a novel predictive model targeted at LBMD: ① Narrow coverage: They exclusively focus on osteoporosis (T-score ≤ − 2.5) and ignore osteopenia (T-score: −1.0 to −2.5)—a preclinical stage of osteoporosis and key component of LBMD—resulting in missed opportunities for early intervention. ② Single-dimensional indicators: Most rely solely on demographic factors (e.g., age, weight) and lack integration of bone metabolism-related biomarkers (e.g., serum creatinine, phosphorus, magnesium) that are readily available in routine physical examinations. Our preliminary analysis confirms these biomarkers are independent risk factors for LBMD (e.g., serum magnesium: OR = 0.006), which can significantly improve screening accuracy. ③ Dependence on specialized equipment: Tools like SCORE still require DXA results for validation, conflicting with the demand for “simple and efficient” screening in primary care settings (especially in rural China).

Against this backdrop, we define LBMD as the combination of osteopenia (*T*-score: −1.0 to −2.5) and osteoporosis (*T*-score ≤ −2.5), based on three lines of scientific evidence: ① Pathological continuity: According to Gourlay et al. (8), osteopenia progresses to osteoporosis over time—with transition periods of 16.8 years (early osteopenia: *T*-score −1.5 to −1.0), 5 years (middle osteopenia: *T*-score −2.0 to −1.5), and 1 year (late osteopenia: *T*-score −2.5 to −2.0)—and untreated osteopenia eventually increases fracture risk. ② Clinical unmet need: Existing tools fail to screen for osteopenia, leaving a large population at risk of unrecognized bone loss. ③ Alignment with research goals: Our study aims to establish an “early prediction model,” and LBMD covers the full spectrum of early bone loss (from initial reduction to osteoporosis), enabling timely intervention.

Epidemiological data further underscores the urgency of LBMD screening in Chinese women (the core population of this study). In 2014, the National Osteoporosis Foundation (NOF) of the United States reported a high prevalence of osteopenia, with approximately 43.4 million people aged 50 years and older diagnosed with osteopenia in 2010 (14). The Korean National Health and Nutrition Examination Survey revealed that the prevalence of osteopenia among postmenopausal women aged ≥ 50 years reached 45.3% (15). In contrast, Chinese data reveal earlier and more severe bone loss in women: a 2018 national epidemiological survey showed that the prevalence of osteopenia in individuals aged 40–49 years was 32.9%, and that in individuals aged 50 years and older was 46.4% (16). This suggests that without timely intervention, individuals with osteopenia will eventually progress to osteoporosis and possibly to severe outcomes such as osteoporotic fractures, with a shorter transition time to OP as bone mass decreases. Therefore, early detection and intervention of osteopenia may reduce the risk of subsequent osteoporosis and even fractures.

The onset of bone loss is insidious. When patients experience clinical symptoms such as pain, fractures, and spinal deformities, severe osteoporosis has already developed, and the best opportunity to prevent and treat osteoporosis is lost. Therefore, it is necessary to find a simple and efficient method to screen for bone loss.

Therefore, we constructed a predictive nomogram for LBMD among Chinese adult women to achieve early, convenient and precise identification of individuals at high risk of bone loss. This model helps clinicians deliver personalized interventions targeted to each woman’s risk profiles at an early stage to reduce her risk of developing osteopenia. For susceptible individuals identified by the model, regular bone mineral density monitoring and timely standardized management are recommended, which may further lower the population-wide incidence of osteoporosis and osteoporotic fractures.

## 2 Methods

### 2.1 Study design

This was a cross-sectional diagnostic prediction model study conducted to screen independent risk factors for LBMD in women and develop a predictive nomogram model with external validation. All data were retrospectively extracted from the hospital electronic medical record (EMR) system covering the period from January 1, 2019, to December 31, 2021.

The training cohort consisted of female examinees who met the predefined inclusion and exclusion criteria from the Health Management Center, Jinshan Campus, the First Affiliated Hospital of Chongqing Medical University. Patients diagnosed with LBMD were classified as the case group, while females with normal bone mineral density served as controls. Predictive factors significantly associated with LBMD identified via multivariate regression analysis were integrated to construct the nomogram. These subjects were randomly divided into training and validation sets at a 7:3 ratio, with a random seed of 12,345,678. Subjects in the training set were screened for relevant risk factors for LBMD and a prediction model for LBMD was constructed. Internal validation was conducted using the internal validation set.

During the identical study period, adult female examinees satisfying the same inclusion and exclusion criteria who attended the outpatient department of Yuanjiagang Campus, the First Affiliated Hospital of Chongqing Medical University, were enrolled as the independent external validation cohort. This separate cohort was used to comprehensively evaluate the discrimination and calibration performance of the established nomogram prediction model. No artificial interventions or prospective follow-ups were performed in this study. The First Affiliated Hospital of Chongqing Medical University Ethics Committee approved the study (2024–254-01). All methods were performed following the relevant guidelines and regulations.

### 2.2 Study subjects

The training set and internal validation set were sourced from the population undergoing physical examinations at the Health Management Center, Jinshan Campus, the First Affiliated Hospital of Chongqing Medical University. The external validation set was obtained from female examinees who visited the outpatient department of Yuanjiagang Campus, the First Affiliated Hospital of Chongqing Medical University during the same period. The validation cohort was collected from a different department. The two campuses are geographically separated, equipped with independent patient admission systems.

### 2.3 Inclusion criteria

1. Female, over 18 years old, with complete clinical data, such as patient’s sex, age, body mass index (BMI), etc.
2. The patient underwent DXA bone densitometer testing.

### 2.4 Exclusion criteria

1. Individuals with concurrent thyroiditis and hyperthyroidism.
2. Those with other bone metabolic disorders, such as rickets, osteomalacia, and osteosclerosis.
3. Patients with severe mental illness.
4. Individuals who were receiving calcium, glucocorticoids, calcitonin, or other drugs known to influence bone metabolism, the concomitant diseases, disease history and medication history of patients were used to determine whether they took related drugs.

### 2.5 Data collection

We conducted a comprehensive review of the literature to identify factors associated with bone loss. Ultimately, the factors we selected included the patient’s sex, age, symptoms, height, BMI, *Helicobacter pylori* stool antigen positivity, systolic blood pressure (SBP), diastolic blood pressure (DBP), blood glucose, cholesterol, low-density lipoprotein (LDL), high-density lipoprotein (HDL), triglycerides, alanine aminotransferase (ALT), aspartate aminotransferase (AST), total bilirubin, total protein, albumin, calcium, phosphorus, magnesium, uric acid, creatinine, urea, platelet count, and hemoglobin (HB). All clinical data including baseline demographic characteristics, laboratory biomarkers, and DXA-derived bone mineral density parameters were retrospectively extracted from the hospital EMR system.

### 2.6 Bone mineral density measurement and standards

Dual-energy X-ray absorptiometry (DXA; Medix DR, France) was used to measure the bone mineral density (BMD, in g/cm^2^) of the lumbar spine (L1–L4) and the left femoral neck for each participant. All DXA scans were performed by certified radiologic technologists following a strict standardized operating procedure to minimize inter-operator variability. Quality control and calibration of the DXA machine were performed daily using a manufacturer-provided phantom to ensure measurement accuracy and consistency (the long-term coefficient of variation was <1.0%). According to the International Society for Clinical Densitometry (ISCD) (17), the diagnosis was based on the lowest T-score among the measured sites referring to WHO criteria (6), The *T*-value of BMD was calculated as follows: T = (measured value − peak BMD)/standard deviation of peak BMD in normal adults. A *T*-value ≤ − 2.5 indicated osteoporosis, *a* − 1 > *T*-value < −2.5 indicated osteopenia, and a *T*-value ≥ − 1 indicated normal BMD. LBMD included osteopenia and osteoporosis.

### 2.7 Development and assessment of the nomogram

In the training set, indicators with statistical significance (*p* < 0.05) from the univariate analysis were combined with collinearity diagnostic (VIF < 10) results and used to select relevant risk factors in the multiple logistic regression analysis (Forward, Conditional). The entry criterion was *p* ≤ 0.05 and the removal criterion was *p* ≥ 0.10. A nomogram was developed based on these independent predictive factors (*p* < 0.05) to predict the occurrence of LBMD in females. Predictor lines were drawn upwards to confirm the points received from the nomogram. The sum of the points for calculating these risk factors was located on the “total score” axis; then, a vertical line was drawn downwards, intersecting the bottom scale to determine the possibility of women suffering from LBMD. Afterwards, the visual prediction model was validated internally and externally. The Hosmer−Leme show test and coefficient of determination (*R*^2^) were used to evaluate the goodness of fit of the model. The ROC curve, AUC and consistency index (C-index) were used to evaluate the predictive accuracy of the model. The calibration curve was used to evaluate the conformity of the model predictions, while decision curve analysis (DCA) reflected the net benefit of the model to patients.

### 2.8 Statistical analysis

This study was a retrospective study on osteoporosis in women. All subjects who met the inclusion and exclusion criteria during the study period were continuously included. Binary logistic regression was used to construct a prediction model for the occurrence of LBMD. Twenty-one independent variables were included for multiple logistic regression to construct the prediction model. The sample size met the principle of EPV (Events Per variable) ≥ 10, that is, the number of LBMD cases ≥21 × 10 = 210 cases. At the same time, the number of cases in the internal validation set and the external validation set were 291 and 381, respectively, which both met the EPV ≥ 10 principle, suggesting that the sample size could meet the needs of modeling and validation. Statistical analyses were performed using IBM SPSS Statistics 25.0 (IBM Corporation, Armonk, NY, United States), including collinearity diagnostics, intergroup comparisons, and logistic regression analysis. ROC curves and nomogram were constructed using R software (version 4.2.2, R Core Team, Vienna, Austria) and MSTATA software^1^ (MStata, Beijing, China). Normality was assessed using the Kolmogorov–Smirnov test, and the two-sample *t*-test was applied for between-group comparisons when data were normally distributed. Non-normally distributed data are presented as medians (P_25_, P_75_), and group comparisons were performed using the Mann–Whitney *U* test. Qualitative data such as HP infection and case detection data are presented as counts (n) and percentages (%), and group comparisons were conducted using the chi-square test.

A nomogram was developed based on the final multiple logistic regression model to predict the risk of low bone mineral density in women, following the standard nomogram construction methodology (18, 19). The point scale for each predictor variable was assigned proportionally to its regression coefficient (*β*-value) in the model, reflecting the variable’s relative contribution to the outcome. The variable with the largest *β*-value was assigned a maximum of 100 points, and the scales of other variables were calibrated proportionally. The total points were calculated by summing the points of each individual variable, and the risk scale was calibrated to convert total points into the predicted probability of low bone mineral density.

The lower and upper limits of the 95% confidence interval for a proportion were calculated using the Wilson method as described by Robert Newcombe in “Two-Sided Confidence Intervals for the Single Proportion: Comparison of Seven Methods” (20). A significance level of *p* < 0.05 was considered to indicate a statistically significant difference.

## 3 Results

### 3.1 Characteristics of the subjects

A total of 3,121 female examinees who underwent health examinations at the Health Management Center, Jinshan Campus, the First Affiliated Hospital of Chongqing Medical University from January 1, 2019 to December 31, 2021 were included in the study. Of these, a random computer number generator selected 70% (*n* = 2,185) into the training cohort and 30% (*n* = 936) into the internal validation cohort. The detailed flow diagram is presented in Figure 1. The average age of the included population was 49.7 ± 11.19 years, with a median age of 50 years, a minimum age of 21 years, and a maximum age of 90 years. The incidence rate of LBMD was 30.66% (957/3121). Among them, the proportions of osteopenia and osteoporosis were 21.08% (658/3121) and 9.58% (299/3121), respectively. The baseline characteristics of the study participants are summarized in Table 1. There was no significant difference (*p* > 0.05) between the training and validation sets, except for creatinine levels (*p* = 0.016) (Table 1). Supplementary Table 3 summarizes the descriptive statistics of all continuous variables for participants allocated to the training and internal validation sets, with data expressed as mean ± standard deviation (SD). In addition, Supplementary Table 4 presents the descriptive statistics of continuous variables stratified by the LBMD and control groups within the training cohort.

**Figure 1:**
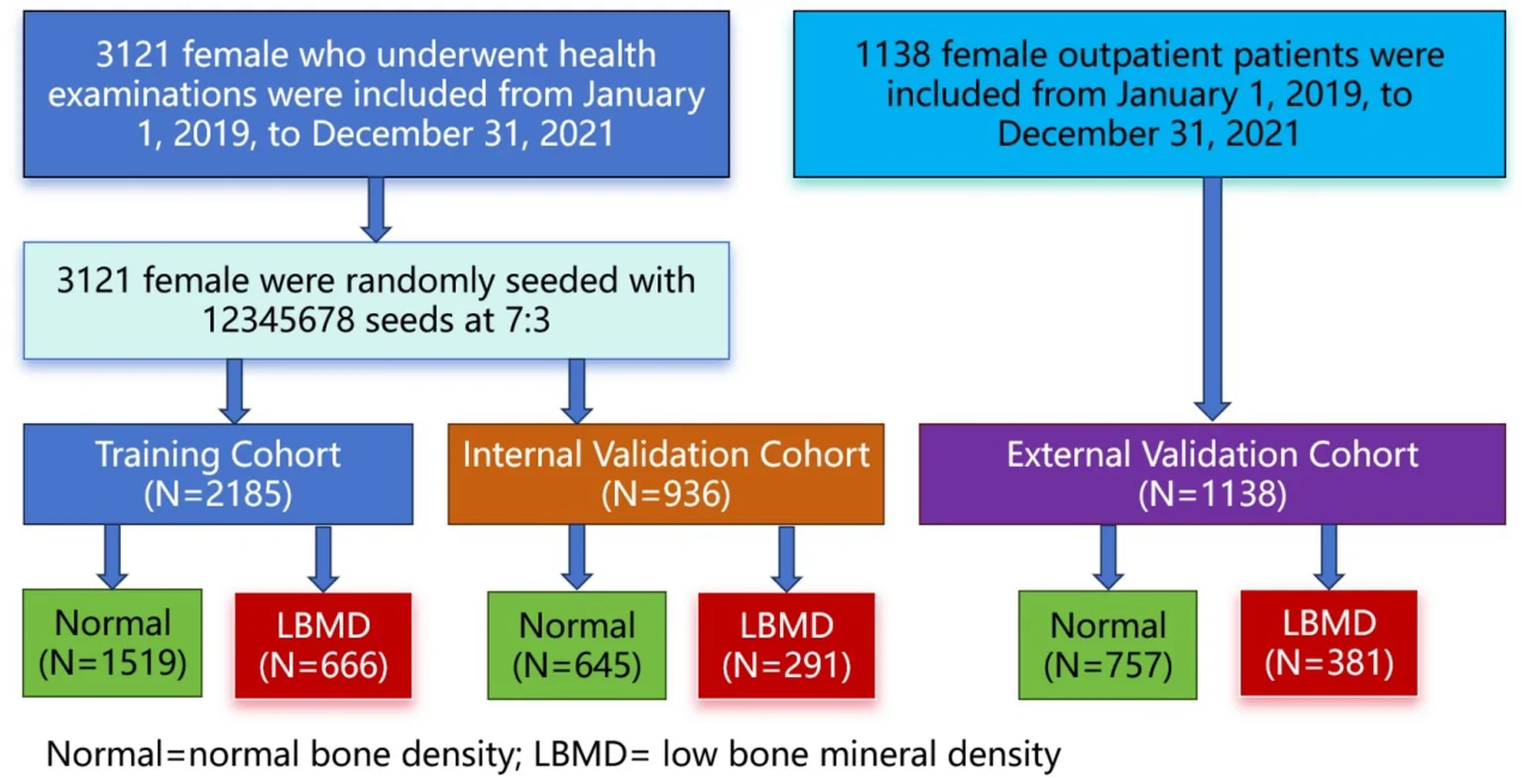
Flow chart subjects.

**Table 1:** Characteristics of the population in the training and internal validation groups.

Characteristic	Total (*N* = 3,121)	Training cohort (*N* = 2,185)	Internal validation cohort (*N* = 936)	*p*-value
	Median (IQR)/*n* (%)	Median (IQR)/*n* (%)	Median (IQR)/*n* (%)	
Age (years)	50 (42, 57)	50 (41, 57)	50 (42, 57)	0.742
Weight (kg)	55 (51, 60)	55 (51, 60)	55 (51, 60)	0.465
Height (cm)	158.0 (154.0, 161.0)	158.0 (154.0, 161.0)	158.0 (154.0, 161.0)	0.943
BMI (kg/m^²^)	22.27 (20.55, 24.35)	22.31 (20.55, 24.39)	22.19 (20.49, 24.31)	0.526
HP (+)	968 (31%)	672 (31%)	296 (32%)	0.631*
SBP (mmHg)	118 (108, 131)	118 (108, 131)	118 (108, 130)	0.789
DBP (mmHg)	70 (63, 77)	70 (63, 78)	69 (63, 77)	0.163
FBG (mmol/L)	5.20 (4.90, 5.50)	5.20 (4.90, 5.50)	5.20 (4.90, 5.50)	0.892
TC (mmol/L)	5.14 (4.51, 5.83)	5.13 (4.51, 5.83)	5.15 (4.53, 5.82)	0.653
LDL (mmol/L)	2.73 (2.12, 3.32)	2.71 (2.10, 3.31)	2.79 (2.18, 3.36)	0.116
HDL (mmol/L)	1.54 (1.34, 1.75)	1.54 (1.34, 1.75)	1.53 (1.33, 1.75)	0.910
TG (mmol/L)	1.10 (0.80, 1.56)	1.10 (0.80, 1.57)	1.08 (0.80, 1.53)	0.388
ALT (U/L)	16 (12, 22)	16 (12, 22)	16 (12, 22)	0.959
AST (U/L)	22 (18, 26)	21 (18, 26)	22 (18, 26)	0.352
TBIL (μmol/L)	17.2 (13.3, 17.9)	17.2 (13.2, 17.9)	17.2 (13.3, 18.0)	0.907
TP (g/L)	74.0 (72.0, 77.0)	74.0 (72.0, 77.0)	74.0 (72.0, 77.0)	0.329
Albumin (g/L)	45.00 (43.00, 46.00)	45.00 (43.00, 46.00)	45.00 (43.00, 46.00)	0.862
Calcium (mmol/L)	2.30 (2.24, 2.37)	2.30 (2.24, 2.37)	2.30 (2.24, 2.37)	0.676
Phosphorus (mmol/L)	1.19 (1.09, 1.28)	1.19 (1.09, 1.28)	1.16 (1.07, 1.26)	0.251
Magnesium (mmol/L)	0.89 (0.85, 0.93)	0.89 (0.85, 0.93)	0.88 (0.84, 0.92)	0.656
Uric acid (μmol/L)	275 (223, 323)	275 (222, 322)	276 (226, 323)	0.441
Creatinine (μmol/L)	57 (52, 62)	56 (51, 62)	57 (52, 63)	0.016
Urea (mmol/L)	4.70 (4.00, 5.60)	4.70 (4.00, 5.50)	4.70 (4.00, 5.60)	0.881
Platelet (10^9^/L)	222 (188, 259)	222 (189, 258)	222 (187, 261)	0.737
HB (g/L)	137 (131, 143)	137 (131, 143)	137 (131, 143)	0.197
LBMD (*n*, %)	957 (30.66)	666(30.48)	291 (31.08)	0.735*

IQR, Interquartile Range; BMI, body mass index; HP, Helicobacter pylori; SBP, systolic blood pressure; DBP, diastolic blood pressure; FBG, fasting blood glucose; TC, total cholesterol; LDL, low density lipoprotein; HDL, high density lipoprotein; TG, triglyceride; ALT, alanine aminotransferase; AST, aspartate aminotransferase; TBIL, total bilirubin; TP, total protein; HB, hemoglobin; LBMD, low bone mineral density. *Pearson’s chi-squared test was applied for categorical variables. Continuous variables with skewed distributions are presented as median (IQR), and between-group comparisons were conducted using the Mann–Whitney *U* test.

### 3.2 Risk factors related to LBMD

The incidence rate of LBMD in the training set was 30.48% (95%CI: 25.59–32.44%) (666/2185). There were significant differences (*p* < 0.05) in age, weight, HP (+), and other factors between the LBMD group and the control group. Other characteristics, such as BMI, SBP, DBP, blood glucose, cholesterol, LDL, triglyceride, ALT and AST activity, total bilirubin, total protein, albumin, serum calcium, serum phosphorus, serum magnesium, uric acid, creatinine, urea, HB, platelet count, and height, were also evaluated. These factors showed varying distributions and *p* values, indicating potential differences between the two groups. Notably, the differences in HDL and total bilirubin levels did not reach statistical significance (*p* = 0.154 and *p* = 0.268, respectively) (Table 2).

**Table 2:** Characteristics of training cohort.

Characteristic	Total (*N* = 2,185)	Control group (*N* = 1,519)	Group with LBMD (*N* = 666)	*p*-value
	Median (IQR)/*n* (%)	Median (IQR)/*n* (%)	Median (IQR)/*n* (%)	
Age (years)	50 (41, 57)	45 (38, 51)	58 (54, 65)	<0.001
Weight (kg)	55 (51, 60)	56 (51, 61)	55 (50, 60)	0.008
Height (cm)	158.0 (154.0, 161.0)	158.0 (155.0, 162.0)	155.0 (151.0, 159.0)	<0.001
BMI (kg/m^²^)	22.31 (20.55, 24.39)	22.07 (20.39, 24.24)	22.74 (21.09, 24.88)	<0.001
HP (+)(*n*, %)	672 (31%)	442 (29%)	230 (35%)	0.011
SBP (mmHg)	118 (108, 131)	114 (106, 127)	126 (115, 139)	<0.001
DBP (mmHg)	70 (63, 78)	69 (62, 76)	74 (67, 80)	<0.001
FBG (mmol/L)	5.20 (4.90, 5.50)	5.10 (4.80, 5.40)	5.30 (5.00, 5.80)	<0.001
TC (mmol/L)	5.13 (4.51, 5.83)	5.02 (4.41, 5.66)	5.41 (4.81, 6.10)	<0.001
LDL (mmol/L)	2.71(2.10–3.31)	2.63 (2.05, 3.22)	2.89 (2.23, 3.50)	<0.001
HDL (mmol/L)	1.54 (1.34, 1.75)	1.54 (1.34, 1.75)	1.52 (1.32, 1.73)	0.154
TG (mmol/L)	1.10 (0.80, 1.57)	1.03 (0.75, 1.46)	1.30 (0.95, 1.80)	<0.001
ALT (U/L)	16 (12, 22)	15 (12, 21)	18 (14, 24)	<0.001
AST (U/L)	21 (18, 26)	21 (17, 25)	23 (20, 28)	<0.001
TBIL (μmol/L)	17.2 (13.2, 17.9)	17.2 (13.2, 17.8)	17.2 (13.3, 18.1)	0.268
TP (g/L)	74.0 (72.0, 77.0)	74.7 (72.0, 77.0)	74.0 (72.0, 77.0)	0.341
Albumin (g/L)	45.00 (43.00, 46.00)	45.00 (43.00, 47.00)	45.00 (43.00, 46.00)	0.039
Calcium (mmol/L)	2.30 (2.24, 2.37)	2.30 (2.23, 2.36)	2.32 (2.26, 2.39)	<0.001
Phosphorus (mmol/L)	1.17 (1.07, 1.27)	1.18 (1.07, 1.27)	1.11 (1.01, 1.21)	<0.001
Magnesium (mmol/L)	0.88 (0.84, 0.92)	0.88 (0.84, 0.92)	0.87 (0.83, 0.91)	0.001
Uric acid (μmol/L)	275 (222, 322)	273 (221, 319)	282 (224, 332)	0.019
Creatinine (μmol/L)	56 (51, 62)	56 (51, 62)	57 (52, 63)	0.033
Urea (mmol/L)	4.70 (4.00, 5.50)	4.60 (3.80, 5.30)	5.10 (4.40, 6.00)	<0.001
Platelet (10^9^/L)	222 (189, 258)	228 (194, 264)	210 (177, 246)	<0.001
HB (g/L)	137 (131, 143)	137 (130, 143)	138 (132, 144)	0.021

IQR, interquartile range; BMI, body mass index; HP, Helicobacter pylori; SBP, systolic blood pressure; DBP, diastolic blood pressure; FBG, fasting blood glucose; TC, total Cholesterol; LDL, low density lipoprotein; HDL, high density lipoprotein; TG, triglyceride; ALT, alanine aminotransferase; AST, aspartate aminotransferase; TBIL, total bilirubin; TP, total protein; HB, hemoglobin; LBMD, low bone mineral density.

After combining factors with *p* < 0.05, professional knowledge, and collinearity test results, we analyzed five factors – age, weight, magnesium, phosphorus, and creatinine – using multiple logistic regression analysis. According to the variance inflation factor (VIF), after excluding the factors with VIF values greater than 10 (BMI), the VIF values of the included factors were all less than 5 (from 1.04 to 3.831), and it was considered that there was no obvious multicollinearity among the variables. These factors were identified as important independent risk factors for LBMD: age (OR = 1.204; 95% CI: 1.184, 1.225, *p* < 0.001), weight (OR = 0.943; 95% CI: 0.927–0.960, *p* < 0.001), serum magnesium (OR = 0.600 per 0.1 mmol/L; 95% CI: 0.486–0.741, *p* < 0.001), serum phosphorus (OR = 0.698 per 0.1 mmol/L; 95% CI: 0.64–0.761, *p* < 0.001), and serum creatinine (OR = 0.984; 95% CI: 0.970–0.999, *p* = 0.034) (Table 3).

**Table 3:** Multivariate binary logistic regression of factors related to low bone mineral density.

Characteristic	*B*	S. E	Wald	*P*	OR	OR 95% CI	
						Lower Limit	Upper limit
Age (years)	0.186	0.009	455.868	0.000	1.204	1.184	1.225
Weight (kg)	−0.059	0.009	43.895	<0.001	0.943	0.927	0.960
Creatinine (μmol/L)	−0.016	0.008	4.508	0.034	0.984	0.970	0.999
Magnesium (0.1 mmol/L)^†^	−0.511	0.108	22.584	<0.001	0.600	0.486	0.741
Phosphorus (0.1 mmol/L)^†^	−0.359	0.044	67.111	<0.001	0.698	0.641	0.761
Constant	2.240	1.213	3.410	0.065	9.395		

S. E, standard error; OR, odd ratio; CI, confidence interval. ^†^ORs for serum magnesium and serum phosphorus are expressed per 0.1 mmol/L increments to reflect clinically meaningful changes; ORs for the other variables are expressed per unit as indicated (age per year, weight per kg, creatinine per μmol/L).

### 3.3 The LBMD prediction equation and nomogram development

Based on the results of the multivariable logistic regression analysis, the LBMD prediction equation was as follows: Logit(*P*) = 2.240 + 0.186 × Age − 3.590 × Phosphorus − 5.111 × Magnesium − 0.016 × Creatinine − 0.059 × Weight. The Cox *R*^2^ and Nagelkerke *R*^2^ in training set were 0.383 and 0.541, respectively. Based on the maximum Youden index, we selected a cut-off value of 0.25774, which balanced the sensitivity and specificity. Additionally, to enhance the sensitivity or specificity of the predictive equation, different cut-off values (0.21820, 0.48198) were tested, and the changes in sensitivity and specificity are presented in Supplementary Table 1.

Each factor associated with LBMD was scored based on its impact. We created a visual nomogram to represent the predictive model (Figure 2). The AUC of the nomogram was 0.894 (95% CI: 0.877–0.912), and the Cox’s *R*^2^ and Nagelkerke *R*^2^ were 0.383 and 0.541 in training set, respectively. The Hosmer–Lemeshow test result showed *p* = 0.258, indicating that the model was sufficiently accurate. In the training set, the calibration plots demonstrated an excellent correlation between the observed and predicted osteopenia (Figure 3A). The results of the ROC curve analyses are displayed in Figure 3. The AUC was 0.894 (95% CI: 0.877–0.912) for the training set (Figure 3A), indicating that the predictive accuracy of the nomogram model was acceptable. The calibration curve of the LBMD prediction model demonstrated favorable statistical concordance between model-predicted risks and observed probabilities in the training cohort, indicating no systematic bias in internal risk estimation (Figure 4A). In addition, DCA showed that the use of the nomogram for predicting the probability of LBMD would result in greater net benefit if the threshold probability was between 5 and 85%, which indicated great potential for screening strategies based on the nomogram for LBMD risk assessment (Figure 5A).

**Figure 2:**
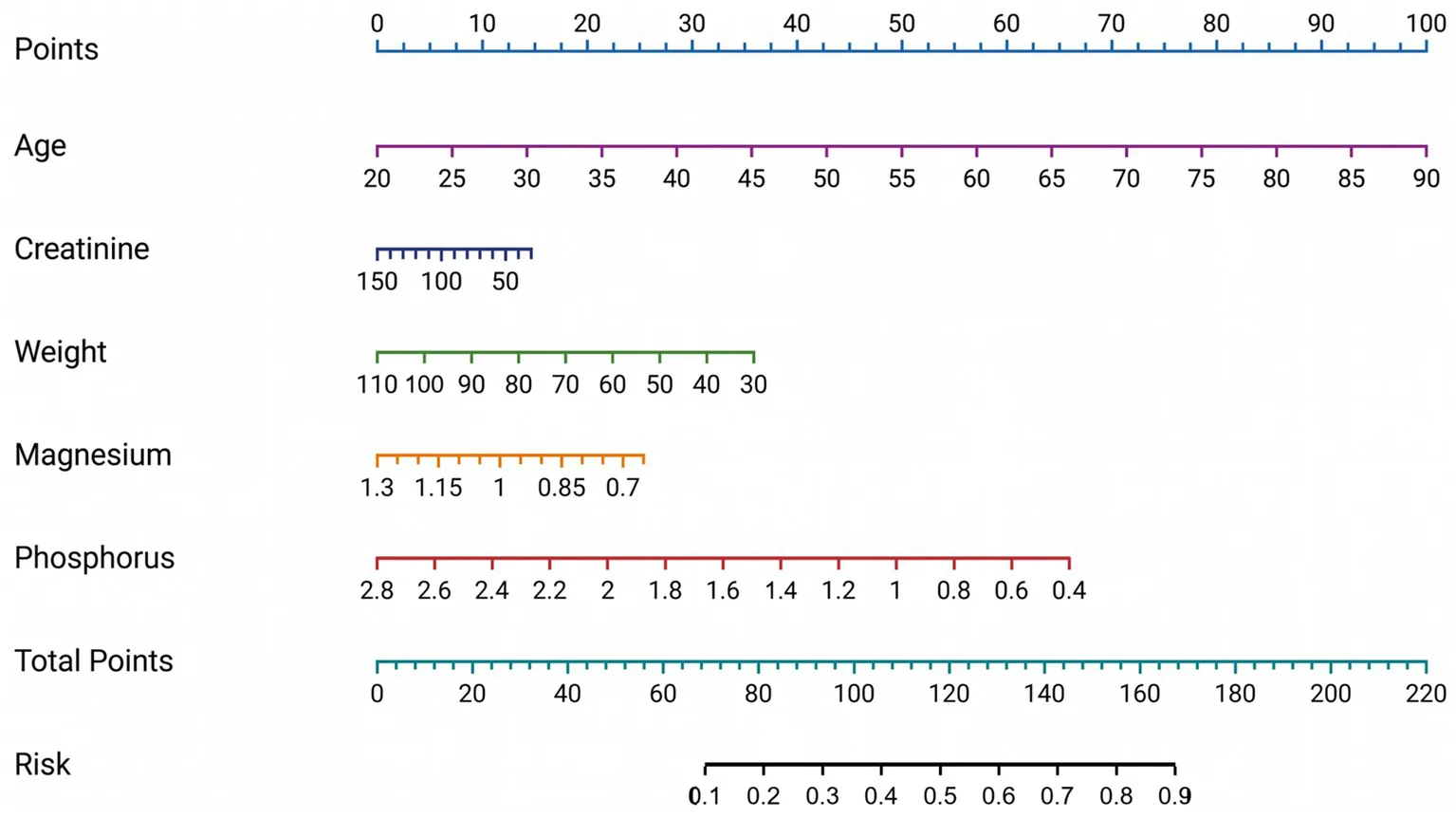
Nomogram for prediction of low bone mineral density in women.

**Figure 3:**
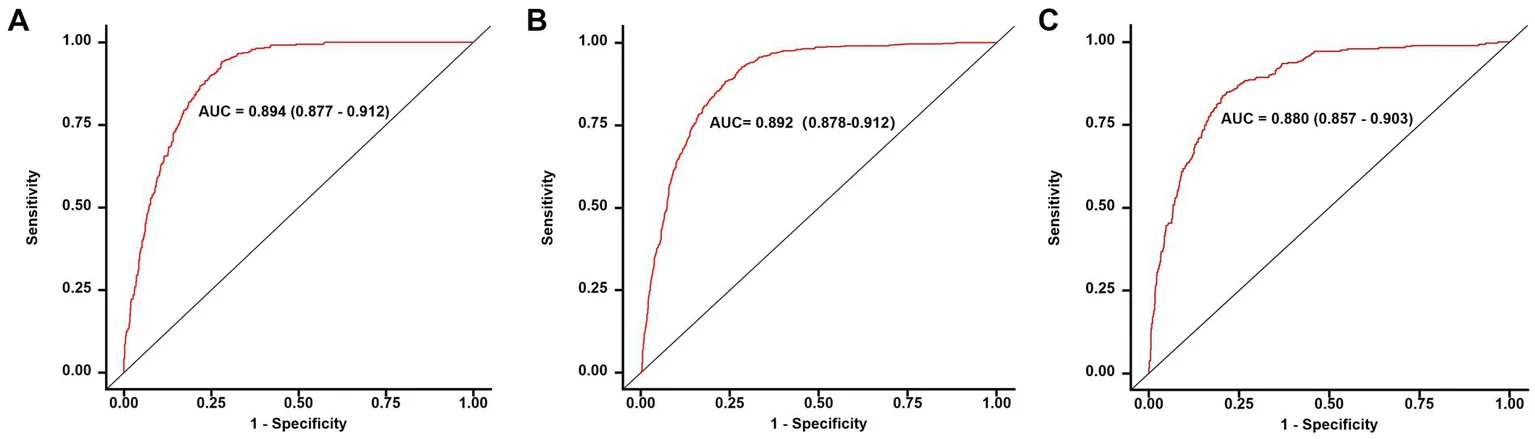
The ROC curve of the prediction model for low bone mineral density. Panel **(A)** based on the training set; panel **(B)** based on internal validation set; panel **(C)** based on external validation set.

**Figure 4:**
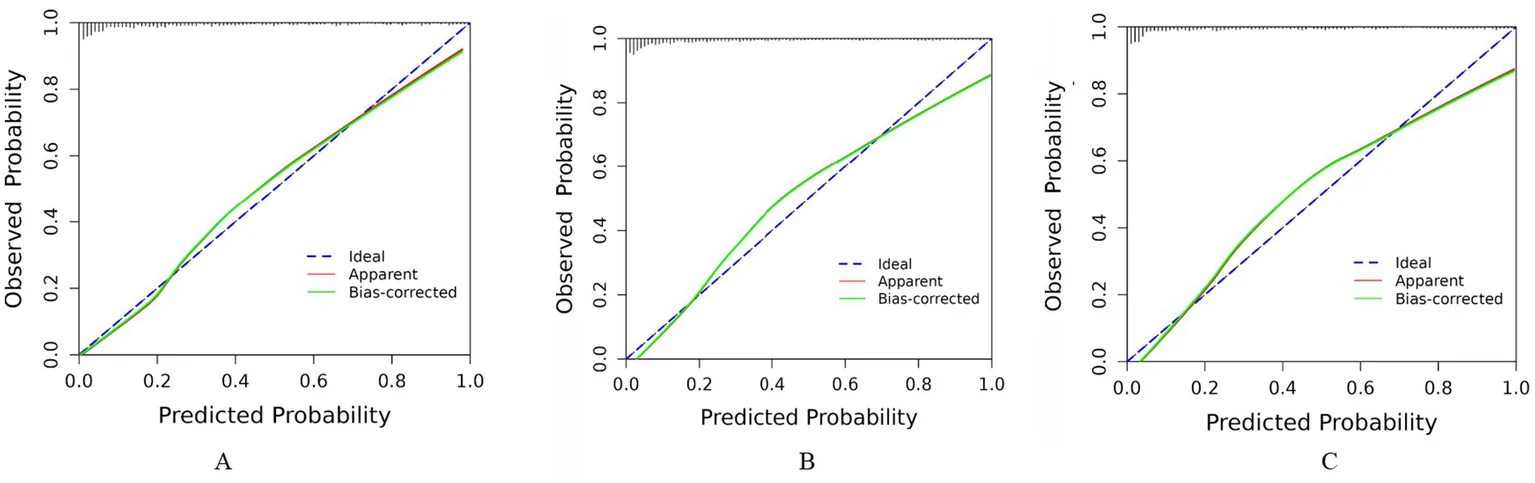
The calibration curve of the prediction model for low bone mineral density. Panel **(A)** based on the training set; panel **(B)** based on internal validation set; panel **(C)** based on external validation set.

**Figure 5:**
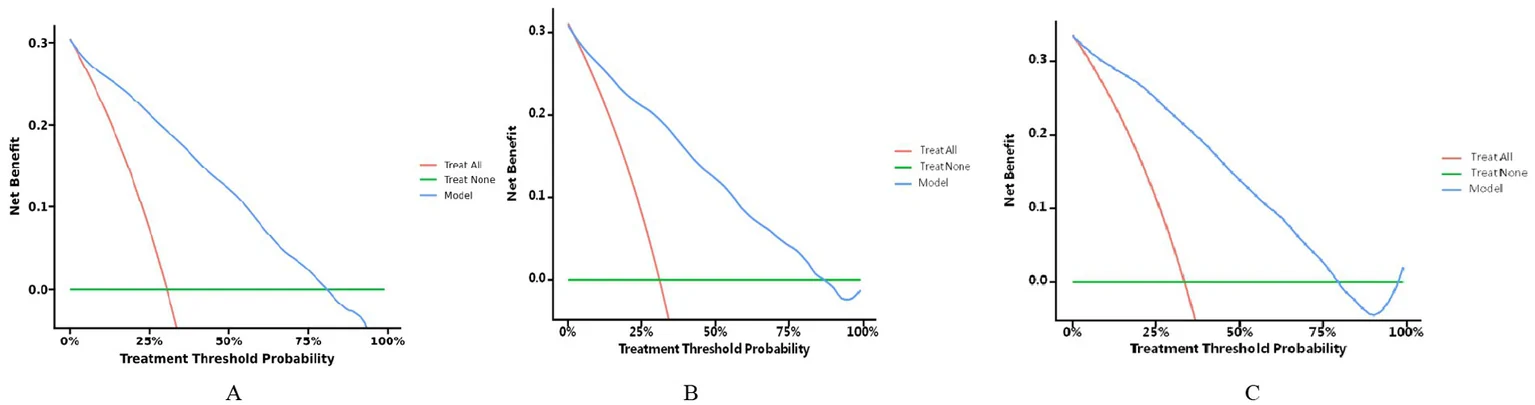
The DCA curve of the prediction model for low bone mineral density. Panel **(A)** based on the training set; panel **(B)** based on internal validation set; panel **(C)** based on external validation set.

### 3.4 Internal and external validation of the predictive models

#### 3.4.1 Internal validation of the predictive model

The internal validation set consisted of 936 individuals, among whom 291 (31.09, 95%CI: 28.21–34.13%) experienced LBMD. The median age of the patients in the internal validation set was 50 (42, 57) years, and there were no statistically significant differences compared to those in the training set (Table 1). The AUC for the internal validation set was 0.892 (95% CI: 0.878–0.905) (Figure 3B), demonstrating high diagnostic performance. The calibration curve of the prediction model showed good predictive accuracy between the actual probability and the predicted probability of LBMD in the internal validation group (Figure 4B). Figure 5B shows the DCA curve based on the internal validation set. The results indicated that if the threshold probability of the patient was between 5 and 85%, the LBMD risk assessment screening strategy based on the column chart would generate greater net benefits.

#### 3.4.2 External validation of the predictive model

We included participants who visited the outpatient department of Yuanjiagang Campus, the First Affiliated Hospital of Chongqing Medical University as an external validation set. Of the 1,345 enrolled participants, 207 were excluded. Overall, 1,138 participants were enrolled. The average age of the external validation cohort was 50 years and the incidence of LBMD was 33.48% (95%CI: 30.80–36.27%) (Supplementary Table 2).

External validation revealed an ROC AUC of 0.880 (0.857–0.903; Figure 3C), which was not significantly different from the ROC AUC of the developed predictive model (*p* = 0.865). Figure 4C was based on an external validation set, and the curve showed that the apparent and bias-corrected curves fit well and were close to the ideal curve. Figure 5C shows the DCA curve based on an external validation set. The results indicated that if the patient’s threshold probability was between 5 and 85%, the screening strategy for LBMD risk assessment based on the nomogram yielded a greater net benefit.

## 4 Discussion

Bone loss is occurring at a higher incidence rate within the population. Surveys report the prevalence of low bone mass in Chinese individuals aged 40 to 49 years to be 32.9%, with 34.4% of men and 31.4% of women affected (21). Despite the large number of osteopenia patients worldwide, tools for predicting LBMD are currently scarce. In this study, we devised and established a nomogram designed for the personalized prediction of LBMD in women. This predictive model is based on common clinically available data, allowing for the quick and convenient prediction of the risk of bone loss in women. This can aid in the early examination of high-risk groups to detect bone loss and enable patients to receive timely and appropriate intervention measures. However, there is still a lack of predictive models for LBMD.

This tool incorporates data such as age, weight, and levels of magnesium, phosphorus, and creatinine to predict the risk of bone loss using routine physical examination results. In our comprehensive study, we scrutinized a spectrum of clinically and statistically significant factors. To devise a streamlined yet potent model, we established a baseline model that focused on fundamental parameters. These parameters — age, weight, phosphorus, magnesium, and creatinine — were chosen based on their pivotal roles in previously developed models. Based on these optimized parameters, we developed a ridge line plot model for the personalized prediction of LBMD in women.

The predictive model developed in this study demonstrated strong potential, with an area under the ROC curve of 0.894 for the training set and commendable scores of 0.892 and 0.880 for internal and external validation, respectively. This performance is supported by clear clinical benchmarks: Hung et al. (22) established a deep learning-based BMD prediction model for women with an AUC of 0.82–0.85, and the OSTA tool recommended by the IOF for osteoporosis screening in Asian women has an AUC of approximately 0.75–0.80 (9, 23). Our model’s AUC is significantly higher than the above-mentioned tools, confirming its superior discriminative ability. Furthermore, the model exhibited robust calibration, as evidenced by favorable Hosmer–Lemeshow test results and calibration curve analyses. This indicates a high degree of accuracy in the model’s predictions.

In the present study, a positive association was found between age and LBMD. A previous study showed that human bone density reaches its peak around the age of 20–29 years, and beyond this point, bone mass and strength gradually decrease every year (24). The accumulation of senescent cells and the acquisition of a senescence-associated secretory phenotype in bone cells, which occurs in almost all tissues, are hallmarks of aging. In a study by Hiremath et al. (25), a 20% increase in the risk of osteoporosis was revealed for every five-year increase in age. The study by Juan et al. (26) demonstrated that the incidence of osteoporosis increases significantly with advancing age in both men and women.

The association between body weight and LBMD remains controversial. In our study, a notable negative correlation existed between LBMD and body weight (OR = 0.943, 95% CI: 0.927–0.960; *p* < 0.001), indicating that an increase in body weight corresponds to an increase in bone density. An appropriate body weight usually implies good nutrition for the patient and indicates a certain level of load on the individual skeleton which is beneficial for the prevention and treatment of bone loss. However, it should be noted that our study did not measure body fat percentage, which has been shown in other studies to be negatively associated with bone density, possibly due to chronic inflammation and adipokine imbalance (27, 28). Future studies should include body composition analysis to further clarify this relationship.

In this study, we found a negative association between phosphorus and LBMD (OR = 0.698 per 0.1 mmol/L, 95% CI: 0.64–0.761; *p* < 0.001). This suggests that lower serum phosphorus levels are associated with higher risk of LBMD, consistent with the role of phosphorus in bone mineralization. Hypophosphatemia can lead to diminished osteoid mineralization, resulting in conditions such as rickets and/or osteomalacia (29). While excessive phosphorus intake has been linked to calcium loss and osteoporosis in some settings (30), our data indicate that in this population, low phosphorus is a more salient risk factor for LBMD.

We found a negative correlation between serum magnesium and LBMD (OR = 0.600 per 0.1 mmol/L; 95% CI: 0.486, 0.741 – *p* < 0.001). Although our study included both premenopausal and postmenopausal women, previous research has highlighted the particular importance of magnesium in postmenopausal populations (31, 32). Future studies should stratify by menopausal status to further elucidate the role of magnesium in different subgroups.

In our study, a mild negative correlation was observed between serum creatinine levels and LBMD (OR = 0.984, 95% CI: 0.970–0.99; *p* = 0.034), This finding contrasts with some studies in populations with renal dysfunction, where creatinine is positively correlated with osteoporosis (33, 34). However, in individuals with normal renal function, higher creatinine may reflect greater muscle mass, which is protective for bone health (35). Our results suggest that within the normal range, creatinine may serve as a proxy for muscle mass rather than renal dysfunction.

Menopausal status is a well-recognized driver of accelerated bone loss in adult women. In the present study, menopausal history was incompletely recorded in our real-world clinical data and could therefore not be entered as a covariate. Considering the close association between chronological age and the timing of menopausal transition, we performed an age-stratified sensitivity analysis in the external validation cohort to examine whether age could serve as a surrogate indicator of ovarian function decline. The prevalence of LBMD was markedly lower in women younger than 50 years (1.76%) than in those aged 50 years or older (59.42%). This pattern is consistent with the biological expectation that estrogen withdrawal around menopause sharply increases skeletal fragility, and supports the interpretation that the model’s age term partly captures the transition to an estrogen-deficient state. Importantly, the model retained acceptable discrimination in both age subgroups (AUC = 0.879 for <50 years; AUC = 0.757 for ≥50 years), indicating that its predictive value is not confined to a single age stratum (Table 4 and Figure 6). Nevertheless, the absence of directly ascertained menopausal status remains a limitation, and future cohorts with complete reproductive history documentation are warranted to refine the model.

**Table 4:** Stratified analyses in the external validation cohort (*N* = 1,138).

Age	*N*	LBMD(*n*,%)	AUC	95%CI lower limit	95%CI upper limit
Total	1,138	381(33.48)	0.880	0.857	0.903
<50岁	512	9(1.76)	0.879	0.800	0.959
≥50岁	626	372(59.42)	0.757	0.719	0.795

AUC, area under the ROC curve; LBMD, Low bone mineral density; CI, confidence interval.

**Figure 6:**
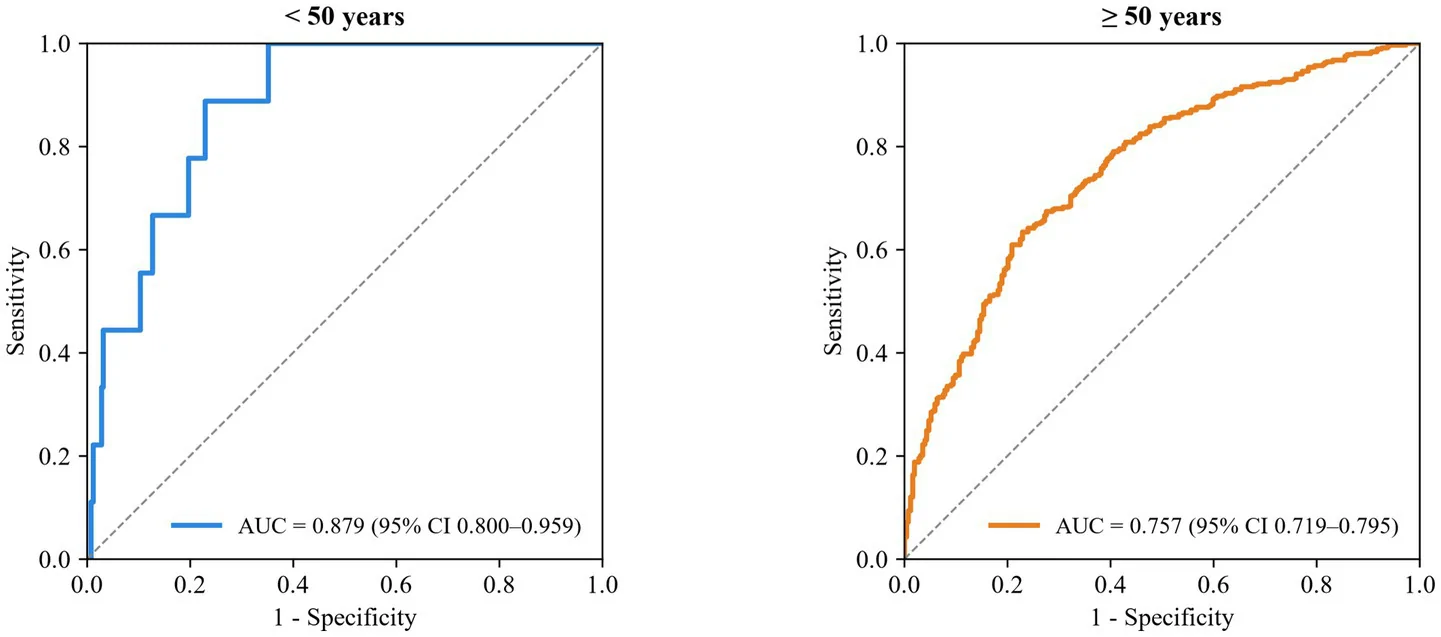
ROC curves of the nomogram for LBMD stratified by age (external validation set, *N* = 1,138).

### 4.1 Advantages and limitations

This study has a large sample size, complete data, and high accuracy of the prediction model. The factors in the LBMD prediction model are general demographics and routine blood test biomarkers. The nomogram is intuitive, simple and suitable for the general population. This study has some limitations. Sex hormone levels are one of the important risk factors for female osteoporosis. Unfortunately, we did not obtain this information from the physical examination population, which becomes one of the factors affecting the accuracy of the prediction model. Additionally, our study population was drawn from a health management center and outpatient department, which may introduce selection bias toward more health-conscious individuals. We did not have complete data on all potential confounders (e.g., dietary intake, physical activity), and missing data were excluded listwise, which may affect generalizability. Therefore, these findings need to be validated and revised in a wider population in the future.

## 5 Conclusion

The incidence of LBMD in Chinese adult women is relatively high. Older age, lower body weight, reduced serum creatinine, reduced serum phosphorus, and reduced serum magnesium were independent risk factors for LBMD in women. The prediction model for LBMD in women established by these factors was statistically validated to have high positive and negative predictive values. Internal and external validation showed that the model has relatively accurate predictive performance. The demographic and blood test indicators of this model are easily obtained in clinical practice and can be conveniently used for risk assessment of individual LBMD patients. This nomogram may help identify high-risk individuals for earlier DXA screening and lifestyle intervention, which could potentially improve outcomes based on existing evidence. However, prospective studies are needed to confirm that its use leads to reduced fracture incidence or improved bone health. In the future, the addition of external validations from different countries and regions could enhance the model’s efficacy and generalizability.

## Data Availability

The original contributions presented in the study are included in the article/Supplementary material, further inquiries can be directed to the corresponding author.

## Footnotes

1
www.mstata.com
