# Supplementary material for: Correlate factors and nomogram model establishment for women with low bone mineral density: a cross-sectional diagnostic prediction model study
Source: Front Public Health. 2026 Oct 9;14:1969424. doi: 10.3389/fpubh.2026.1969424 (PMC13693812; doi:10.3389/fpubh.2026.1969424)
Supplement: Supplementary file 1 [file Data_Sheet_1.docx]

Supplementary Table 1 The impact of different cut-off values in a predictive equation on sensitivity and specificity

|  | **Cut off=0.25774**  **Balancing sensitivity and specificity** | | | **Cut off=0.21820**  **Improved sensitivity** | | | **cut off=0.48198**  **Improved specificity** | | |
| --- | --- | --- | --- | --- | --- | --- | --- | --- | --- |
|  | **Training set** | **Internal validation set** | **External validation set** | **Training set** | **Internal validation set** | **External validation set** | **Training set** | **Internal validation set** | **External validation set** |
| **Sen（%,95CI）** | 88.44  （85.79-90.65） | 87.63  （83.35-87.63） | 90.81  （87.49-93.32） | 91.89  （89.57-93.73） | 89.35  （85.28-92.40） | 95.80  （93.29-97.4） | 63.81  （60.09-67.37） | 63.57  （57.90-68.89） | 66.40  （61.51-70.96） |
| **Spe（%,95CI）** | 76.1  （73.89-78.18） | 74.26  （70.75-77.48） | 72.72  （69.43-75.78） | 71.96  （69.65-74.16） | 69.15  （65.48-72.59） | 69.27  （65.89-72.46） | 90.06  （88.45-91.47） | 89.3  （86.68-91.46） | 86.36  （83.73-88.62） |
| **+PV（%,95CI）** | 61.87  （58.74-64.90） | 60.57  （55.83-65.12） | 62.68  （58.57-66.61） | 58.96  （55.94-61.91） | 56.64  （52.07-61.10） | 61.14  （57.17-64.97） | 73.78  （70.04-77.21） | 72.83  （67.05-77.93） | 71.07  （66.15-75.54） |
| **-PV（%,95CI）** | 93.76  （92.27-94.98） | 93.01  （90.47-94.91） | 94.01  （91.78-95.66） | 95.29  （93.91-96.37） | 93.5  （90.92-95.38） | 97.03  （95.23-98.16） | 85.02  （83.19-86.68） | 84.46  （81.55-86.99） | 83.59  （80.83-86.02） |
| **Acc（%,95CI）** | 79.86  （78.13-81.49） | 78.42  （75.67-80.94） | 78.79  （76.32-81.07） | 78.03  （76.25-79.72） | 75.43  （72.57-78.08） | 78.17  （75.68-80.48） | 82.06  （80.40-83.61） | 81.30  （78.68-83.67） | 79.67  （77.23-81.91） |
| **YI** | 0.645 | 0.619 | 0.635 | 0.638 | 0.585 | 0.651 | 0.539 | 0.529 | 0.528 |

Sen, sensitivity; Spe, specificity; +PV, positive predict value; -PV, negative predict value, Acc, accuracy, Youden, Youden index.

Supplementary Table 2 Characteristics of the population in training and internal validation cohort and external validation cohort

| **Characteristic** | **Training and internal validation Cohort**  **(N =3121)** | **External Validation Cohort**  **(N = 1138)** | **p-value** |
| --- | --- | --- | --- |
|  | **Median (IQR)/n (%)** | **Median (IQR)/n (%)** |  |
| **Age (years)** | 50 (42, 57) | 51 (41, 58) | 0.072 |
| **Weight (kg)** | 55 (51, 60) | 56 (51, 61) | 0.375 |
| **Height (cm)** | 158.0 (154.0, 161.) | 157.0 (153.0, 161.0) | 0.007 |
| **BMI (%)** | 22.27 (20.55, 24.35) | 22.49 (20.66, 24.84) | 0.014 |
| **HP (+)** | 968 (31%) | 301 (26%) | 0.004 |
| **SBP (mmHg)** | 118 (108, 131) | 119 (109, 132) | 0.078 |
| **DBP (mmHg)** | 70 (63, 77) | 70 (63, 78) | 0.351 |
| **FBG (mmol/L)** | 5.20 (4.90, 5.50) | 5.20 (4.90, 5.50) | 0.945 |
| **TC (mmol/L)** | 5.14 (4.51, 5.83) | 5.23 (4.58, 5.92) | 0.027 |
| **LDL (mmol/L)** | 2.73 (2.12, 3.32) | 2.87 (2.37,3.41) | <0.001 |
| **HDL (mmol/L)** | 1.54 (1.34, 1.75) | 1.55 (1.33, 1.79) | 0.181 |
| **TG (mmol/L)** | 1.10 (0.80, 1.56) | 1.10 (0.81, 1.57) | 0.988 |
| **ALT (U/L)** | 16 (12, 22) | 14 (10, 20) | <0.001 |
| **AST(U/L)** | 22 (18, 26) | 18 (15, 21) | <0.001 |
| **TBIL (mmol/L)** | 17.2 (13.3, 17.9) | 17.3 (14.4, 20.9) | <0.001 |
| **TP (mmol/L)** | 74.0 (72.0, 77.0) | 74.0 (72.0, 77.0) | 0.731 |
| **Albumin (mmol/L)** | 45.00 (43.00, 46.00) | 46.00 (44.00, 47.00) | <0.001 |
| **Calcium (mmol/L)** | 2.30 (2.24, 2.37) | 2.38 (2.33, 2.45) | <0.001 |
| **Phosphorus (mmol/L)** | 1.19 (1.09, 1.28) | 1.21 (1.11, 1.28) | <0.001 |
| **Magnesium (mmol/L)** | 0.89 (0.85, 0.93) | 0.83 (0.80, 0.87) | <0.001 |
| **Uric acid (mmol/L)** | 275 (223, 323) | 297 (255, 341) | <0.001 |
| **Creatinine (μmol/L)** | 57 (52, 62) | 60 (54, 66) | <0.001 |
| **Urea (mmol/L)** | 4.70 (4.00, 5.60) | 4.90 (4.10, 5.70) | <0.001 |
| **Platelet (10^9^/L)** | 222 (188, 259) | 220 (183, 256) | 0.041 |
| **HB (g/L)** | 137 (131, 143) | 136 (130, 141) | 0.002 |
| **LBMD** | 957(30.66) | 381 (33.48%) | 0.086 |

BMI, body mass index; HP, Helicobacter pylori; SBP, systolic blood pressure; DBP, diastolic blood pressure; FBG, Fasting blood glucose; TC, Total Cholesterol; LDL, Low density lipoprotein; HDL, High density lipoprotein; TG, Triglyceride; ALT, Alanine aminotransferase; AST, Aspartate aminotransferase; TBIL, Total bilirubin; TP, Total protein; HB, hemoglobin; LBMD, Low bone mineral density.

Supplementary Table 3 Continuous variable characteristics of the population in training and internal validation cohort (Mean ± SD)

| **Characteristic** | **Total**  **(N =3121)** | **Training Cohort**  **(N = 2,185)** | **Internal Validation Cohort**  **(N = 936)** |
| --- | --- | --- | --- |
| **Age (years)** | 49.7±11.19 | 49.69±11.23 | 49.73±11.1 |
| **Weight (kg)** | 56.12±7.64 | 56.17±7.72 | 56±7.45 |
| **Height (cm)** | 157.35±7.4 | 157.27±8.04 | 157.52±5.64 |
| **BMI (%)** | 22.64±2.98 | 22.67±3.02 | 22.58±2.89 |
| **SBP (mmHg)** | 120.64±17.37 | 120.7±17.47 | 120.47±17.15 |
| **DBP (mmHg)** | 70.94±10.55 | 71.13±10.61 | 70.52±10.39 |
| **FBG (mmol/L)** | 5.36±1.16 | 5.37±1.21 | 5.34±1.02 |
| **TC (mmol/L)** | 5.21±0.98 | 5.21±0.99 | 5.21±0.94 |
| **LDL (mmol/L)** | 2.72±0.95 | 2.71±0.94 | 2.76±0.96 |
| **HDL (mmol/L)** | 1.55±0.33 | 1.55±0.33 | 1.56±0.32 |
| **TG (mmol/L)** | 1.34±1.01 | 1.35±1 | 1.33±1.03 |
| **ALT (U/L)** | 19.43±13.25 | 19.55±13.74 | 19.13±12.05 |
| **AST(U/L)** | 22.91±8.48 | 22.95±8.8 | 22.84±7.67 |
| **TBIL (mmol/L)** | 16.51±5.23 | 16.47±5.12 | 16.61±5.46 |
| **TP (mmol/L)** | 74.53±4 | 74.47±4.13 | 74.65±3.67 |
| **Albumin (mmol/L)** | 44.92±2.54 | 44.93±2.57 | 44.91±2.48 |
| **Calcium (mmol/L)** | 2.31±0.1 | 2.31±0.11 | 2.31±0.1 |
| **Phosphorus (mmol/L)** | 1.18±0.15 | 1.18±0.15 | 1.19±0.15 |
| **Magnesium (mmol/L)** | 0.89±0.06 | 0.89±0.06 | 0.89±0.06 |
| **Uric acid (mmol/L)** | 258.64±102.62 | 257.6±103.72 | 261.08±100.03 |
| **Creatinine (μmol/L)** | 57.33±8.62 | 57.09±8.58 | 57.9±8.68 |
| **Urea (mmol/L)** | 4.81±1.19 | 4.81±1.19 | 4.82±1.19 |
| **Platelet (10^9^/L)** | 225.53±56.34 | 225.38±56.61 | 225.89±55.74 |
| **HB (g/L)** | 136.02±11.25 | 136.04±11.52 | 135.97±10.59 |

BMI, body mass index; SBP, systolic blood pressure; DBP, diastolic blood pressure; FBG, Fasting blood glucose; TC, Total Cholesterol; LDL, Low density lipoprotein; HDL, High density lipoprotein; TG, Triglyceride; ALT, Alanine aminotransferase; AST, Aspartate aminotransferase; TBIL, Total bilirubin; TP, Total protein; HB, hemoglobin; LBMD, Low bone mineral density.

Supplementary Table 4 Continuous variable characteristics of the control group and the LBMD group in the training set (Mean ± SD)

| **Characteristic** | **Total**  **(N = 2,185)** | **Control group**  **（N=1519）** | **LBMD Group**  **（N=666）** |
| --- | --- | --- | --- |
| **Age (years)** | 49.69±11.23 | 45.36±9.7 | 59.56±7.67 |
| **Weight (kg)** | 56.17±7.72 | 56.58±7.92 | 55.25±7.16 |
| **Height (cm)** | 157.27±8.04 | 158.34±7.75 | 154.83±8.16 |
| **BMI (%)** | 22.67±3.02 | 22.53±3.06 | 23±2.88 |
| **SBP (mmHg)** | 120.7±17.47 | 117.4±15.99 | 128.25±18.33 |
| **DBP (mmHg)** | 71.13±10.61 | 69.7±10.26 | 74.37±10.7 |
| **FBG (mmol/L)** | 5.37±1.21 | 5.27±1.02 | 5.59±1.53 |
| **TC (mmol/L)** | 5.21±0.99 | 5.09±0.94 | 5.49±1.05 |
| **LDL (mmol/L)** | 2.71±0.94 | 2.64±0.91 | 2.87±0.99 |
| **HDL (mmol/L)** | 1.55±0.33 | 1.56±0.32 | 1.54±0.33 |
| **TG (mmol/L)** | 1.35±1 | 1.25±0.85 | 1.57±1.25 |
| **ALT (U/L)** | 19.55±13.74 | 18.77±13.94 | 21.35±13.09 |
| **AST(U/L)** | 22.95±8.8 | 22.11±8.99 | 24.85±8.04 |
| **TBIL (mmol/L)** | 16.47±5.12 | 16.37±5.06 | 16.69±5.27 |
| **TP (mmol/L)** | 74.47±4.13 | 74.51±4.15 | 74.39±4.08 |
| **Albumin (mmol/L)** | 44.93±2.57 | 45.01±2.6 | 44.76±2.49 |
| **Calcium (mmol/L)** | 2.31±0.11 | 2.3±0.11 | 2.32±0.11 |
| **Phosphorus (mmol/L)** | 1.18±0.15 | 1.17±0.15 | 1.21±0.16 |
| **Magnesium (mmol/L)** | 0.89±0.06 | 0.88±0.06 | 0.9±0.06 |
| **Uric acid (mmol/L)** | 257.6±103.72 | 255.42±101.64 | 262.58±108.23 |
| **Creatinine (μmol/L)** | 57.09±8.58 | 56.8±8.49 | 57.74±8.75 |
| **Urea (mmol/L)** | 4.81±1.19 | 4.65±1.15 | 5.2±1.17 |
| **Platelet (10^9^/L)** | 225.38±56.61 | 231.25±56.26 | 212±55.15 |
| **HB (g/L)** | 136.04±11.52 | 135.49±12.12 | 137.28±9.92 |

BMI, body mass index; SBP, systolic blood pressure; DBP, diastolic blood pressure; FBG, Fasting blood glucose; TC, Total Cholesterol; LDL, Low density lipoprotein; HDL, High density lipoprotein; TG, Triglyceride; ALT, Alanine aminotransferase; AST, Aspartate aminotransferase; TBIL, Total bilirubin; TP, Total protein; HB, hemoglobin; LBMD, Low bone mineral density.
